# Carotid Endarterectomy Ameliorates Cognitive Impairment in Clinical and Experimental Unilateral Carotid Artery Stenosis

**DOI:** 10.1161/JAHA.124.038388

**Published:** 2025-01-16

**Authors:** Yijun Cheng, Bin Chen, Miao Zhang, Zhenghong Chen, Mingjian Liu, Ziqian Zhang, Hao Tang, Dapeng Wang, Wenwen Lv, Biao Li, Yuting Dai, Hanbing Shang

**Affiliations:** ^1^ Department of Neurosurgery, Center of Pituitary Tumor, Ruijin Hospital Shanghai Jiao Tong University School of Medicine Shanghai China; ^2^ Department of Neurosurgery, Ruijin Hospital Shanghai Jiao Tong University School of Medicine Shanghai China; ^3^ Department of Neurosurgery, Ruijin Hospital Luwan Branch Shanghai Jiao Tong University School of Medicine Shanghai China; ^4^ Department of Nuclear Medicine, Ruijin Hospital Shanghai Jiao Tong University School of Medicine Shanghai China; ^5^ Department of Emergency, Ruijin Hospital Shanghai Jiao Tong University School of Medicine Shanghai China; ^6^ Department of Neurosurgery, Huashan Hospital, Shanghai Medical College Fudan University Shanghai China; ^7^ Department of Neurosurgery, Xuanwu Hospital Capital Medical University Beijing China; ^8^ Clinical Research Center, School of Medicine Shanghai Jiao Tong University Shanghai China; ^9^ Shanghai Institute of Hematology, State Key Laboratory of Medical Genomics, National Research Center for Translational Medicine, Ruijin Hospital Shanghai Jiao Tong University School of Medicine Shanghai China; ^10^ Department of Neurosurgery, Ruijin‐Hainan Hospital Shanghai Jiao Tong University School of Medicine Shanghai Hainan China

**Keywords:** carotid artery stenosis, carotid endarterectomy, cognitive impairment, genomic sequencing, PET/MR, white blood cell, Animal Models of Human Disease, Basic Science Research, Biomarkers, Inflammation, Ischemia

## Abstract

**Background:**

Carotid endarterectomy (CEA) is widely used to treat carotid artery stenosis (CAS). However, the effects of CEA on unilateral CAS‐induced cognitive impairment and the underlying mechanism remain poorly understood.

**Methods and Results:**

Thirteen patients diagnosed with unilateral severe CAS underwent pre‐ and post‐CEA assessments, including ^18^fluoro‐2‐deoxy‐d‐glucose positron emission tomography/magnetic resonance imaging, cognitive assessments, and routine blood tests before and after CEA. Unilateral carotid common artery occlusion and ligation release (reperfusion) surgeries were performed in mice to mimic CAS and CEA. Cognitive function, cerebral blood flow, and white matter damage were evaluated in mice using the Morris water maze test, Doppler flowmetry, laser‐speckle imaging, diffusion tensor imaging, Luxol fast blue staining, transmission electron microscopy, and western blot assays post unilateral carotid common artery occlusion and reperfusion. Genomic sequencing of the white matter was performed to explore the potential underlying mechanism. CEA significantly enhanced the Montreal Cognitive Assessment scores in patients with CAS and preoperative cognitive impairment. Moreover, CEA led to notable improvements in cerebral blood flow, energy metabolism, and white matter integrity, while concurrently reducing blood inflammation. In the mouse model, reperfusion surgery alleviated cognitive deficits, increased cerebral blood flow, and alleviated white matter damage following unilateral carotid common artery occlusion. Furthermore, transcriptional surveys have revealed substantial alterations in the upregulation of Nrf2 signaling and metabolic pathways, coupled with the inhibition of neuroinflammation, cellular communication, and immune cell population signaling following reperfusion.

**Conclusions:**

CEA ameliorated CAS‐induced cognitive dysfunction by improving the cerebral functional structure. These beneficial effects may be attributed to their antioxidant and anti‐inflammatory properties.

**Clinical Perspective**

**What Is New?**

- Carotid endarterectomy (CEA) significantly enhanced the Montreal Cognitive Assessment scores in patients with carotid artery stenosis and preoperative cognitive impairment.
- Hybrid ^18^fluoro‐2‐deoxy‐d‐glucose positron emission tomography/magnetic resonance imaging that contains 3‐dimensional arterial spin labeling and diffusion tensor imaging revealed that CEA led to notable improvements in cerebral blood flow, energy metabolism, and white matter integrity.
- Using the mice unilateral carotid common artery occlusion model, our mechanistic investigations revealed substantial alterations in the upregulation of antioxidant signaling and metabolic pathways, coupled with the inhibition of neuroinflammation, cellular communication, and immune cell population signaling following CEA‐mimic operation.

**What Are the Clinical Implications?**

- CEA can ameliorate carotid artery stenosis‐induced cognitive dysfunction by improving the cerebral functional structure.
- The neuroprotective effects by CEA may be attributed to the antioxidant and anti‐inflammatory properties.
- The white blood cell activation may contribute to cognitive progression in patients with carotid artery stenosis and Montreal Cognitive Assessment‐related cognitive impairment, which can be effectively alleviated by CEA.

**Nonstandard Abbreviations and Acronyms**
^18^F‐FDG: ^18^fluoro‐2‐deoxy‐d‐glucose
CAS: carotid artery stenosis
CBF: cerebral blood flow
CEA: carotid endarterectomy
CI: cognitive impairment
CN: cognitive normal
DTI: diffusion tensor imaging
MMSE: Mini‐Mental State Examination
MoCA: Montreal Cognitive Assessment
SUV: standardized uptake value
UCCAO: unilateral carotid common artery occlusion
WMD: white matter damage

Carotid artery stenosis (CAS) arises primarily from atherosclerotic plaque formation at the common carotid artery bifurcation, involving the origin of the internal carotid artery (ICA) and carotid bulb.^1^ As a significant contributor to chronic cerebral hypoperfusion (CCH), CAS is implicated in approximately 7% of ischemic stroke cases^2^ and is one of the leading causes of vascular cognitive impairment (CI), including vascular dementia.^3^, ^4^ Notably, patients with CAS often manifest notable impairments in executive function, word production, verbal and visual memory, and motor speed,^5^ thereby contributing to substantial socioeconomic burdens and public health challenges.

Carotid endarterectomy (CEA) has emerged as the gold standard treatment for severe (>70%) and symptomatic CAS following NASCET (North American Symptomatic Carotid Endarterectomy Trial) in 1991.^6^ Subsequently, the efficacy of CEA for asymptomatic CAS has gained wide acceptance, with randomized trials indicating a significant absolute risk reduction of approximately 5% to 6% compared with nonsurgical management.^7^, ^8^ Patients benefit from this carotid revascularization procedure, which can be evaluated based on clinical presentation, imaging, and laboratory diagnostic information. However, the impact of CEA on cognitive function with unpredictable outcomes remains controversial.^9^ Conflicting research findings have reported instances of no change, improvement, or even decline in cognitive function following CEA.^10^

The pathophysiological cerebral changes and mechanisms underlying CAS after CEA remain poorly understood. CAS‐induced chronic hypoperfusion, which is shown as decreased cerebral blood flow (CBF) and metabolism, leads to white matter deterioration. As CEA can revascularize the internal carotid artery (ICA), which theoretically is able to ameliorate the hypoperfusive structural dysfunction. Prior studies indicated impaired CBF and white matter structure in Alzheimer disease, which presents as progressive cognitive dysfunction.^11^ Notably, CBF directly affects the cerebral metabolism. However, the changes in CBF, white matter, and metabolism before and after CEA remain unclear. Nowadays, positron emission tomography/magnetic resonance imaging (PET/MRI) has become an effective and accurate method for cerebral functional examination. Although hybrid PET/MRI systems offer a promising approach by combining PET with the advantages of MR over computed tomography (CT) for brain imaging, such as detailed anatomical characteristics and improved cerebral tissue contrast,^12^ their use in studying functional brain alterations in CAS has been limited. In this study, we employed hybrid ^18^fluoro‐2‐deoxy‐d‐glucose (^18^F‐FDG) PET/MRI to evaluate the effects of CEA surgery on brain functional alterations in patients with unilateral severe CAS. Furthermore, we established unilateral CAS animal models and replicated the CEA procedure to evaluate cognitive function, neuropathological changes, and underlying molecular mechanisms in vivo, aiming to shed light on the intricate mechanisms underlying CAS and its response to surgical intervention.

## METHODS

The data that support the findings of this study are available from the corresponding author upon reasonable request. Sequencing data can be viewed in NODE (http://www.biosino.org/node) via the accession ID OEP005154. Code used in the paper is available on GitHub (https://github.com/NRCTM‐bioinfo/CAE_UCCAO_proj_2024).

### Patients and Cognitive Assessment

In this study, 13 patients with CAS were included from 2018 to 2020. Inclusion criteria were patients diagnosed with unilateral CAS whose stenosis ≥70% on CT angiography or digital subtraction angiography examinations. The degree of ICA stenosis was measured as follows by the NASCET (North American Symptomatic Carotid Endarterectomy Trial) criterion, which has been widely used and already harmonized across modalities.^6^

Stenosis (%) = (Diameter of the normal distal ICA − narrowest ICA diameter in the stenotic segment)/diameter of the normal distal ICA × 100.

All patients signed an informed consent form. The exclusion criteria were (1) declining to participate; (2) previous stroke; (3) chronic atrial fibrillation, paroxysmal atrial fibrillation, or heart failure within 6 months; (4) myocardial infarction within 30 days; (5) unstable angina; (6) major psychiatric disease or neurological disease other than transient ischemic attack or stroke; (7) major somatic illness; and (8) poor compliance. This study was approved by the Ethical Review Board of Ruijin Hospital, Shanghai Jiao Tong University School of Medicine (ID: PETMRCEA, no. 20240306094400981).

All patients underwent molecular imaging with ^18^F‐FDG PET/MRI, neurological and cognitive examinations, and CEA measurement. Cognitive function was evaluated using the Mini‐Mental State Examination (MMSE) and Montreal Cognitive Assessment (MoCA) Chinese Version before CEA and 4 days, 6 months, and 12 months after CEA. The MoCA mainly includes the Visuospatial/Executive, Naming, Memory, Attention, Language, Abstraction, Delayed Recall, and Orientation tests. The MMSE includes tests of Temporal orientation, Place orientation, Immediate memory, Delayed memory, Attention, Numeracy, and Language ability. The MMSE and MoCA were performed by an experienced technician who was blinded to the study. Preoperative MMSE ≥27 and MoCA score ≥26 are defined as cognitive normal (CN), and MMSE <27 and MoCA score <26 are defined as CI.

### CEA Operation

All CEA procedures were performed by an experienced neurosurgeon (Prof. Hanbing Shang) under general anesthesia with an operating microscope. During the surgery, brain oxygen and neurophysiological monitoring were performed simultaneously.

### Blood Routine Test Examination

After overnight fasting, blood was harvested in the morning using violet vacuum tubes with EDTA before CEA and 1 year after CEA. Blood samples were sent for routine examination on the same day of collection.

### Hybrid PET/MR Imaging and Analysis

Hybrid PET/MRI (Siemens Healthcare, Erlangen, Germany) studies were performed and reviewed by 2 board‐certified specialists who were blinded to the clinical data. The CBF, standardized uptake value (SUV), and fractional anisotropy (FA) were recorded. The SUV was used for semiquantitative PET analysis, which refers to the radioactive activity of the PET tracers (^18^F‐FDG) in the targeted tissues. The mean of SUV (SUV_mean_) of the referenced regions including the hypoperfusive areas, bilateral prefrontal lateral, prefrontal medial, sensorimotor, anterior cingulate, posterior cingulate, precuneus, parietal superior, parietal inferior, occipital lateral, primary visual, temporal lateral, temporal mesial, cerebellum whole, and pons. The hypoperfusive areas indicate the region with the lowest metabolism on PET/MR imaging. Herein, FA rather than other diffusion tensor imaging (DTI) metrics, such as mean diffusivity, relative anisotropy, and volume ratio, was chosen due to its validity and reliability as an indicator for white matter injury with high sensitivity in cerebral ischemic studies.^13^ Briefly, the scanning procedures were as follows: repetition time/echo time1/echo time 2 (TR/TE1/TE2): 4.0/1.1/2.2 ms; field of view 50 × 37.5 cm; matrix 256 × 128; slice thickness/overlap: 5.2/2.6 mm; 120 image/slab; imaging time: 18 seconds. Patients with CAS received 196 ± 2 MBq ^18^F‐FDG (Medi‐Physics, Nihon, Tokyo, Japan) via an intravenous injection. PET data were obtained and stored in Digital Imaging and Communications in Medicine as 4 × 5 minutes dynamic time frames, 80 to 100 minutes post injection. The PET images were then reconstructed using an iterative reconstruction procedure (3 iterations, 21 subsets) and a Gaussian filter (4 mm). For the MR‐based scatter correction, PET and 3‐dimensional (3D) Dixon volumetric‐interpolated breath‐hold examination sequences were acquired simultaneously. The values of SUV_mean_ and SUV_max_ were calculated using MATLAB software (MathWorks, Natick, MA, USA) to quantify the biodistribution of ^18^F‐FDG. For 3D arterial spin labeling (ASL) elevating CBF, the parameters were applied as follows: 36 slices, postlabeling delay=2.5 seconds, TE=10.7 ms, TR=5335 ms, voxel size=1.88×1.88×4.00 mm^3^, and scanning time=5:10 minutes. DTI data were acquired using DTI studio v.2.4 to calculate the FA after diffusion‐weighted image reconstruction.

### Animals

C57BL/6 mice (6–8 weeks, male, 15–23 g) were purchased from the Experimental Animal Center of the Chinese Academy of Sciences (Shanghai, China). Mice were housed under specific pathogen‐free conditions and provided with sterilized food and water ad libitum. All efforts were made to reduce the total number of mice used and minimize suffering. A total of 75 mice were randomly divided into 3 groups: (1) sham group; (2) unilateral carotid common artery occlusion (UCCAO) group; and (3) reperfusion group (n=25 in each group). All animal experiments were approved by the Institutional Animal Care and Use Committee of the Ruijin Hospital, Shanghai Jiao Tong University School of Medicine.

### Mice UCCAO Surgery and Carotid Artery Ligature Loosen (Reperfusion) Surgery

UCCAO surgery was performed to mimic CAS as previously described with minor modifications.^14^ Briefly, mice were anesthetized with 4% chloral hydrate solution (0.1 mL/10 g; Sigma‐Aldrich, St. Louis, MO, USA) intraperitoneally. The mice were fixed, and their necks were exposed. A longitudinal incision was made on the neck to expose the trachea, which could serve as a marker of the carotid sheath location. The common carotid artery and vagus nerve were separated from each other in the sheath. The right common carotid artery was ligated using 5‐0 silk sutures. Subsequently, the skin on the neck was sutured. Thirty days after UCCAO, the mice were anesthetized again, and a neck incision was made to expose the common carotid artery ligated with silk sutures. Ligation lines were cut to free the common carotid artery and induce blood reperfusion. Thus, carotid artery ligature loosening surgery was performed to mimic CEA. Several days after the ligature loosening surgery, the neuroimaging and biochemical experiments were conducted in each mice.

### Mice CBF Measurement

In vivo infrared Doppler flowmeter and laser speckle imaging (RWD, Shenzhen, Guangdong, China) were used to determine the CBF in each group. Briefly, mouse heads (4 mice from each group) were fixed on a stereotactic head fixator, and the midline scalp was cut to expose the skull. The average baseline CBF for 1 minute was determined as the reference value, and changes in CBF for 5 minutes postoperatively were also recorded. The data were expressed as the percentage of the relative fold of the reference CBF.

### Mice DTI Measurement

All mice underwent DTI 4 weeks after the operation. The mice (4 mice from each group) were anesthetized by isopentane inhalation. The examination was performed using a Bruker (biospec70/20 USR, Germany) 160 mm aperture 7.0T MR scanner and surface coil scanning. First, the location map was collected and the animals were placed. A fast spin‐echo sequence (TE/TR=24.5/2000 ms, field of view=20 mm×20 mm, layer=10, layer thickness=1 mm, matrix=128×128, and acquisition time=2 minutes) was used to obtain the T2WI image. The image acquisition time was 5 minutes. DTI raw data were scanned by the fast spin‐echo‐dual sequence, TE/TR=24.5/2000 ms, field of view=20 mm×20 mm, layer=10, layer thickness=1 mm, Δ=13.72 ms, duration=5 ms, matrix=128×128, B=650 s/mm^2^. The image‐acquisition time was 1 hour.

### Luxol Fast Blue Staining

For pathological evaluation, mouse brains were fixed in paraformaldehyde buffer solution. These paraffin embedded sections were cut, washed with 95% ethanol, and then cultured in luxol fast blue (Merck, Darmstadt, Germany) at 56 °C for 12 hours. Subsequently, the sections were placed in the lithium carbonate solution for 15 seconds to distinguish white matter from gray matter. Finally, the tissues were washed 3 times with distilled water and 80% ethanol and sealed with fresh xylenol. The myelin sheaths were observed under a light microscope. Four mice were employed in each group.

### Transmission Electron Microscope Observation

The mice brain tissues were collected, and fixed in 4 °C precooled phosphate buffered saline solution and 2.5% glutaraldehyde immediately. After that, the white matter tissues were cut into small pieces (1 mm^3^) and then fixed in 2.5% glutaraldehyde at 4 °C for 2 hours and 1% osmic acid for another 2 hours. After dehydration with graded alcohol concentrations, the blocks were made transparent with pure acetone and embedded in neutral resin. An ultrathin slicer was used to cut the blocks into ultrathin sections. The slices were then placed in a copper mesh and stained with uranium acetate/lead citrate. The ultrastructure of the white matter was observed using Hitachi HF‐3300 transmission electron microscope (Chiyoda‐ku, Tokyo, Japan). Four mice were used in each group.

### Western Blot Assay

White matters from different groups (*n*=3) were collected, homogenized, and dissolved in radioimmunoprecipitation assay buffer containing a phosphatase and protease inhibitor cocktail (Millipore, Bedford, MA, USA). Protein concentrations were determined using a bicinchoninic acid protein quantitative kit (Beyotime, Nantong, Jiangsu, China) according to the manufacturer's instructions. A total of 20 μg proteins were resolved with sodium dodecyl sulfate‐polyacrylamide gel electrophoresis on 6% and 15% polyacrylamide gel, and then transferred to polyvinylidene difluoride membranes (Millipore, Temecula, CA, USA). After sealing with 10% milk for 2 hours, the membranes were incubated with the primary antibodies at 4 °C overnight and secondary antibodies (Table S1) for 1 hour at room temperature. Finally, the proteins were visualized by chemiluminescence and the signal intensity was quantified using ImageJ 1.52a software (NIH, Bethesda, MD, USA).

### Morris Water Maze

The Morris water maze test was performed 4 weeks after reperfusion. All procedures were performed as described previously.^15^ Briefly, continuous training was performed for 5 days to enable each mouse to find the hidden platform in the water. When the mice were habituated, the platform was removed on day 6. The procedures of pretraining and subsequent swimming without platform for 1 minute enable mice to habituate the environment and eliminate anxiety in Morris water maze. Also, mice with visual impairment were excluded. The tracking system recorded the driving distance, driving time, and number of times crossing the target quadrant in 1 minute from 6 mice selected randomly from each group.

### RNA‐Seq

To analyze the RNA‐seq data, we aligned the reads to the mouse reference genome GRCm39 (Release 34). The reference genome and annotation file were obtained from the GENECODE database (https://www.gencodegenes.org/mouse/). Alignment was performed using STAR (version 2.7.9a).^16^ Subsequently, RSEM (version 1.3.1)^17^ was used to generate read counts, transcripts per kilobase of exon model per million mapped reads, and fragments per kilobase of transcript per million mapped read metrics for all samples. Differentially expressed genes (DEGs) between different conditions were identified using the R package limma.^18^ The significance level for defining DEGs in the RNA‐seq analysis was set at an adjusted *P* value <0.05, with a log2 fold change >0.26 (fold change 1.2) for upregulated genes and an adjusted *P* value <0.05, with a log2 fold change <−0.26 for downregulated genes. Three mice were employed in each group.

### Functional Enrichment Analysis

Gene symbols between human and mouse genes were transformed using biomaRt (https://github.com/grimbough/biomaRt). Gene set enrichment analysis was performed for functional enrichment analysis in the R package clusterProfiler^19^ using a preranked algorithm. The gene sets used in gene set enrichment analysis were obtained from MSigDB.^20^ Enrichment *P* values were calculated based on 1000 permutations and subsequently adjusted using the Benjamini–Hochberg method. The minimum and maximum sizes of each gene set used in the analysis were set to 5 and 1000, respectively. A significance cutoff was applied, with values of *P* value <0.05 and an adjusted *P* value <0.05 indicating significance.

### Statistical Analysis

SPSS 25.0S software (SPSS Inc., Chicago, IL, USA) and GraphPad Prism 8.0 (GraphPad Software, San Diego, CA, USA) were used to analyze the results in the present study. Normality of data distribution was assessed using the Shapiro–Wilk test. For comparisons of continuous variables among multiple groups, 1‐way ANOVA was employed, followed by the Student–Newman–Keuls post hoc test for pairwise comparisons. For comparisons between 2 independent groups, the unpaired Student's *t* test was used for normally distributed data. Paired *t* test was employed in surgery‐induced values of 2 variables in a single case. Correlations were assessed using Pearson's correlation coefficients. All quantitative data were expressed as the mean±SD. Statistical significance was set at *P*<0.05.

## RESULTS

### CEA Improved Cognitive Function in Patients With CAS

**Figure 1:**
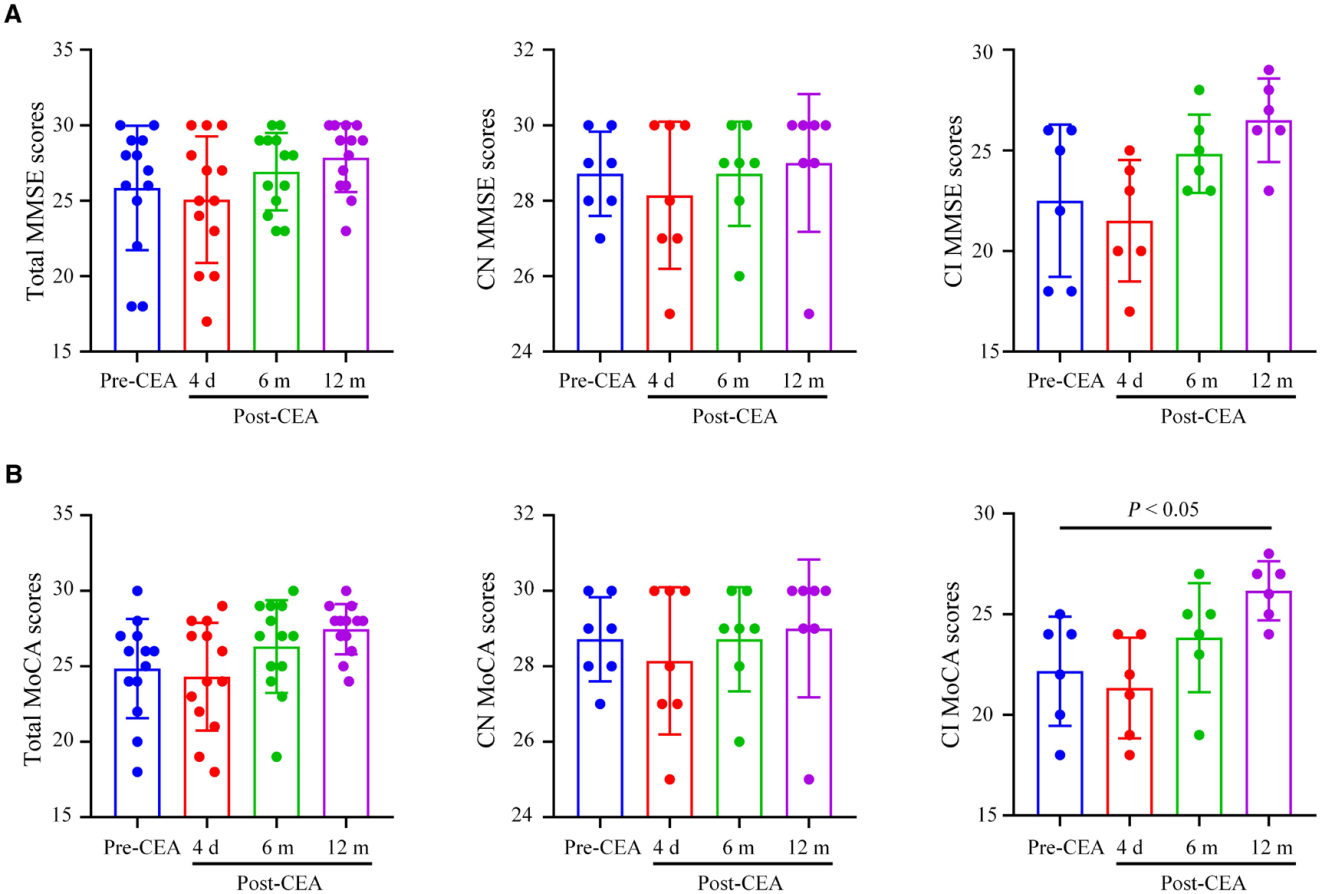
The effects of CEA on cognitive function in patients with CAS. **A**, MMSE scores of total patients (n=13), patients with CN (n=7), and patients with CI (n=6) with CAS before and 4 d, 6 mo, and 12 mo after CEA. **B**, MoCA scores of total patients (n=13), patients with CN (n=7), and patients with CI (n=6) with CAS before and 4 d, 6 mo, and 12 mo after CEA. n=13. The *P* values were calculated using ANOVA. CAS indicates carotid artery stenosis; CEA, carotid endarterectomy; CI, cognitive impairment; CN, cognitive normal; MMSE, Mini‐Mental State Examination; and MoCA, Montreal Cognitive Assessment.

The characteristics of all 13 patients are presented in Table, including 12 men and 1 woman, with ages ranging from 62 to 85 years. To investigate the impact of CEA on cognitive function, we assessed changes in MMSE and MoCA scores pre‐ and postoperatively (Table S2). As depicted in Figure 1A, MMSE scores exhibited a declining trend on postoperative day 4 but showed increasing trends at 6 and 12 months, with no significant difference observed between patients pre‐ and post‐CEA, regardless of whether they had preoperative CI (*P*>0.05) (Figure 1A). The MoCA scores were not significantly different between pre‐CEA and at 4 and 6 months after CEA. However, MoCA scores were significantly elevated at 12 months after CEA compared with the pre‐CEA scores in the group with CI (Figure 1B, *P*<0.05). These findings suggest that CEA may effectively enhance cognitive function in patients with CAS and preoperative cognitive impairment as assessed using the MoCA method.

**Table 1:** Characteristics of 13 Patients With CAS

Patients	Sex	Age, y	Mo of onset	Hypertension	Diabetes	Hypercholesterolemia	Smoking	Alcohol	Education, y	Preoperative symptom	Postoperative symptom	Location of CAS	Stenosis degree	Follow‐up (mo) after diagnosis	Note
1	M	66	120	Y	Y	Y	Y	Y	6	Hemiparalysis	None	R	80%	63	…
2	M	85	84	Y	N	Y	Y	N	9	TIA	None	R	70%	62	…
3	M	62	12	N	Y	N	N	Y	14	Weakness in left leg	None	R	95%	16	Die of COVID‐19
4	F	48	5	N	N	N	N	N	12	Left upper limb numbness	None	R	95%	61	…
5	M	66	6	Y	N	Y	N	N	14	PE	None	L	75%	60	…
6	M	70	1	Y	N	N	Y	Y	12	Right limb numbness	Recurrent laryngeal nerve injury	L	70%	57	…
7	M	75	0.5	Y	N	Y	Y	Y	6	Right upper limb numbness; speech disorders	None	L	90%	54	…
8	M	69	2	N	N	N	N	N	15	Left limb numbness; TIA	None	R	95%	53	…
9	M	64	5	Y	N	N	Y	Y	0	PE	None	R	75%	53	…
10	M	62	1	N	N	N	N	N	12	Dizzy	None	L	90%	53	…
11	M	71	6	Y	N	N	N	N	12	Lower limb numbness; Dizzy	None	R	90%	23	Die of bladder cancer
12	M	74	4	N	N	N	Y	N	16	Dizzy	None	R	85%	52	…
13	M	67	0.3	Y	N	N	Y	N	4	Dizzy	None	R	90%	52	…

CAS indicates carotid artery stenosis; F, female; L, left; M, male; N, no; PE, physical examination; R, right; TIA, transient ischemic attack; and Y, yes.

### CEA Improved Cerebral Functional Structure in Patients With CAS

**Figure 2:**
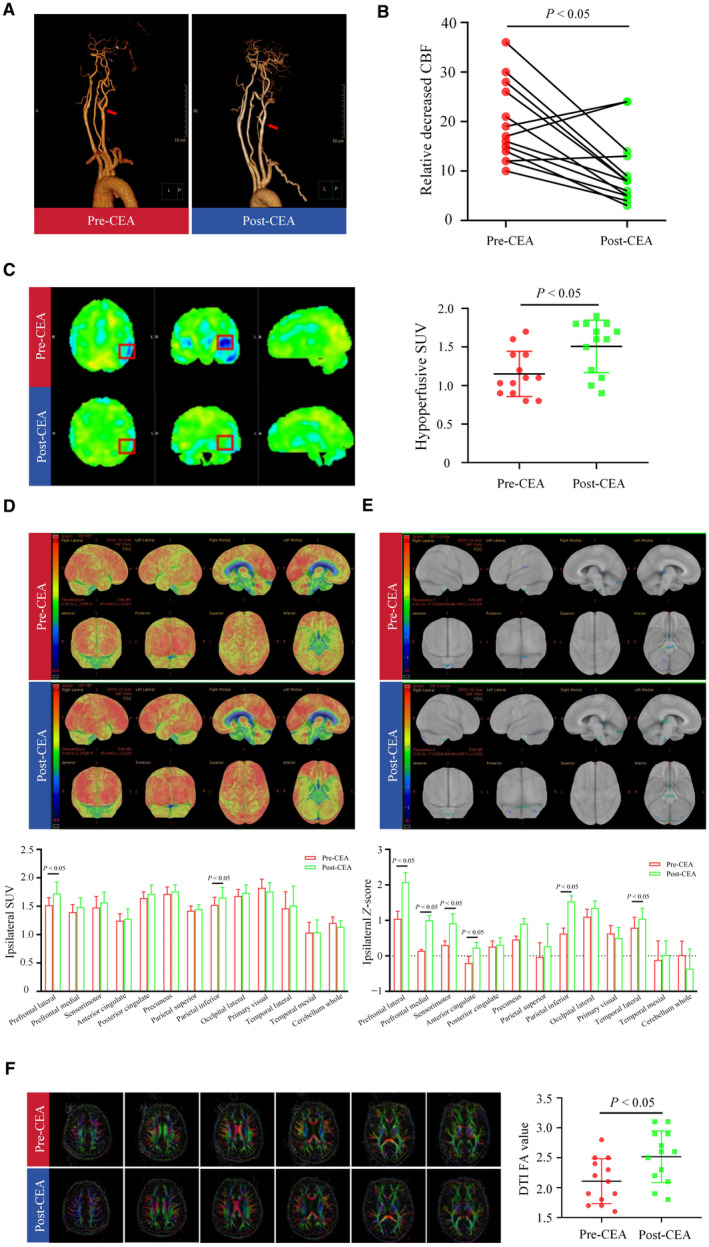
The effects of CEA on brain functional structures in patients with CAS. **A**, Representative imaging of pre‐ and postoperative CTA from a 70‐y‐old man with symptomatic stenosis of the right ICA (Patient #6). **B**, Decreased relative CBF ratio in the hypoperfusion areas before and after CEA. **C**, **Left:** Representative images of pre‐ and postoperative PET/MR from a 67‐y‐old man with symptomatic stenosis of the right ICA (Patient #13); **Right:** SUV_mean_ data before and after CEA in the hypoperfusion areas. **D**, **Upper:** Representative images of pre‐ and postoperative PET/MR from a 70‐y‐old man with symptomatic stenosis of the right ICA (Patient #6); **Lower:** SUV_mean_ data before and after CEA in the whole ipsilateral brains. **E**, **Upper:** Representative images of pre‐ and postoperative *Z* score normalized PET/MR on Patient #6; **Lower**: *Z* score data before and after CEA in the whole ipsilateral brains. **F**, **Left:** Representative images of pre‐ and postoperative DTI from a 66‐y‐old man with symptomatic stenosis of the right ICA (Patient #1); **Right:** FA value data before and after CEA in the hypoperfusion areas. n=13. The *P* values were calculated using Student's *t* test and ANOVA. CAS indicates carotid artery stenosis; CBF, cerebral blood flow; CEA, carotid endarterectomy; CTA, computed tomography angiography; DTI, diffusion tensor imaging; FA, fractional anisotropy; ICA, internal carotid artery; PET/MR, positron emission tomography/magnetic resonance; SUV, standardized uptake value; and WMD, white matter damage.

To directly observe the functional changes in the brain after CEA, we used a hybrid ^18^F‐FDG PET/MR system. As depicted in Figure 2A, CEA effectively widened the narrowed ICA, as shown on CT angiography imaging. The preoperative decreased relative CBF ratio (compared with the contralateral side) in the hypoperfusion areas was significantly improved after CEA at 6 months using MRI 3D ASL imaging (*P* < 0.05, Figure 2B). Moreover, ^18^F‐FDG PET revealed a notable increase in tracer uptake in the hypoperfusion areas in the postoperation group compared with the preoperation group, with an SUV_mean_ of 1.15 preoperative versus 1.51 postoperative (*P*<0.05, Figure 2C). Additionally, as for the whole brain, CEA induced a significant glucose metabolic elevation in the ipsilateral prefrontal lateral and parietal inferior regions (*P*<0.05, Figure 2D). Furthermore, the *Z* score was used for metabolic data normalization in different brain regions. As shown in Figure 2E, the *Z* scores in the ipsilateral prefrontal‐lateral, prefrontal‐medial, sensorimotor, anterior cingulate, parietal inferior, and temporal lateral regions were significantly elevated following CEA (*P*<0.05). Moreover, CEA improved the contralateral prefrontal lateral, prefrontal medial, and prefrontal lateral metabolism (*P*<0.05, Figure S1A,B). To detect changes in white matter damage (WMD) after CEA, we performed DTI of white matter fibers by measuring the FA value. As shown in Figure 2F, white matter morphology was disordered and rarefied preoperatively; however, these injuries were alleviated following CEA. Quantitatively, CEA significantly improved the FA values in the ipsilateral hemisphere at 6 months (*P*<0.05), highlighting its potential therapeutic benefits in improving cerebral function and integrity following carotid artery stenosis. We further investigated the potential correlations between neuroimaging and cognitive measures at 6 months after CEA; only CEA‐induced changes of MMSE scores were positively correlative with CBF (*P*<0.05, Figure S2).

### Reperfusion Operation Improved the Cognitive Function In Vivo

**Figure 3:**
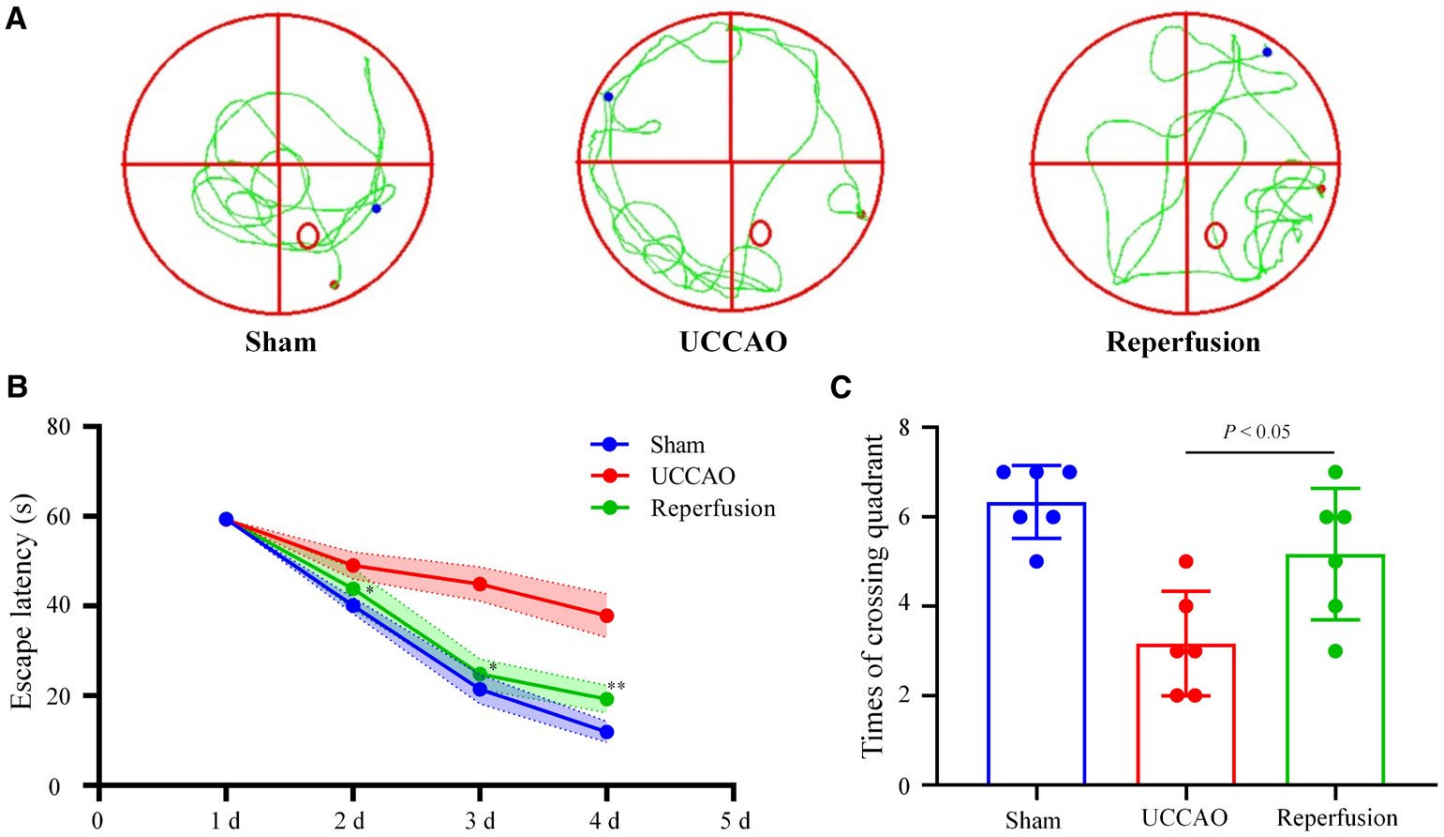
The effects of reperfusion surgery on cognition in UCCAO mice. **A**, Representative images of MWM test in sham, UCCAO, and reperfusion groups. **B**, Escape latency scores of mice from different groups on days 1 to 4 post training. **C**, Times of swimming across the correct quadrant of mice from different groups on day 5 post training. n=6. The *P* values were calculated using ANOVA. MWM indicates Morris water maze; and UCCAO, unilateral carotid common artery occlusion.

To further verify the cognition‐protective effects of CEA on CAS, we established CAS mice models using UCCAO surgery, followed by reperfusion surgery to simulate CEA by loosening the carotid artery ligature. Subsequently, we performed the Morris water maze test in vivo (Figure 3A). From days 1 to 4 post training, reperfusion surgery led to a significant reduction in the UCCAO‐induced decline in escape latency scores (all *P*<0.05, Figure 3B). Moreover, the mice in the reperfusion group had significantly longer swimming times across the correct quadrant than mice in the UCCAO group on day 5 post training (*P*<0.05, Figure 3C).

### Reperfusion Operation Increased CBF and Alleviated WMD In Vivo

**Figure 4:**
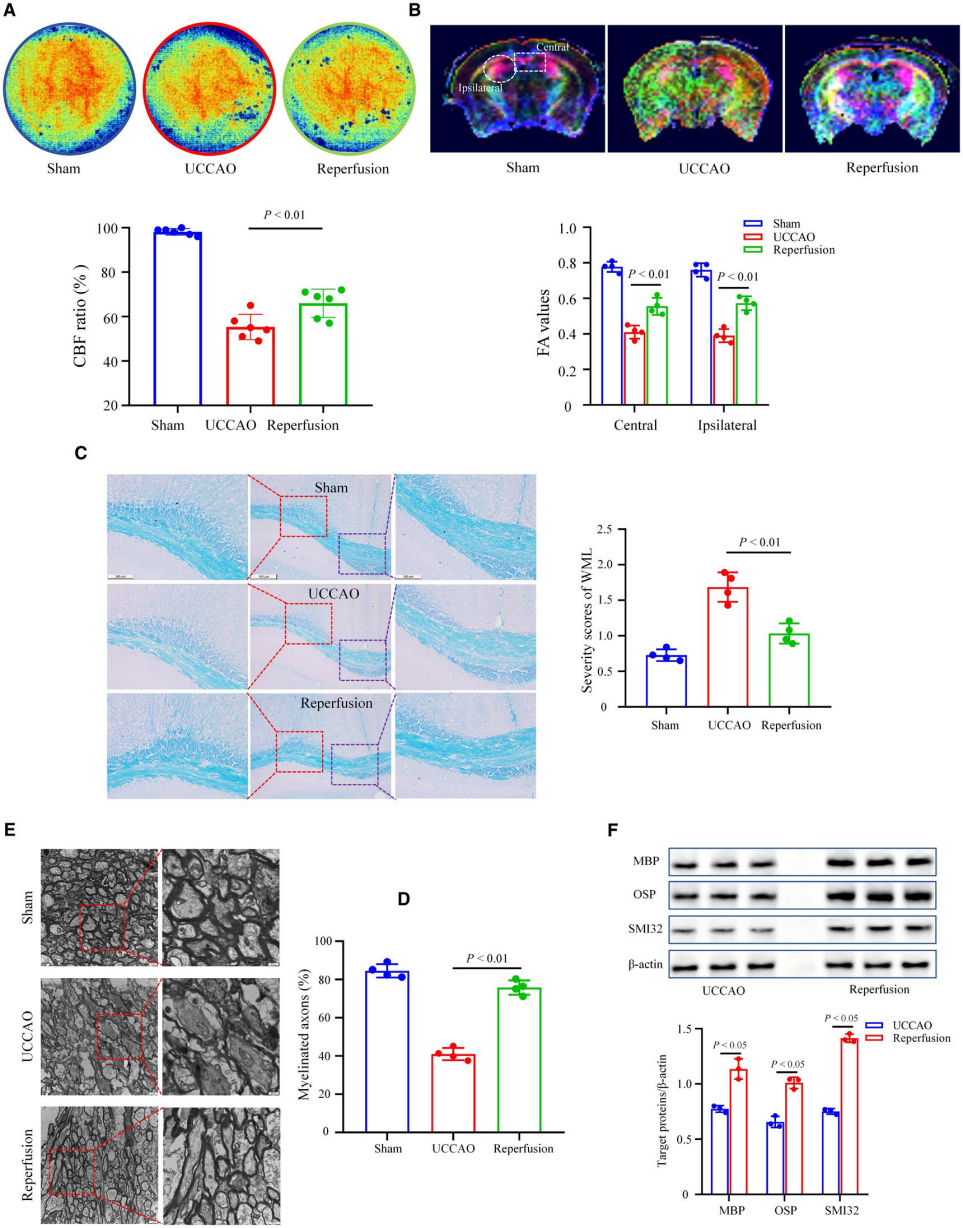
The effects of reperfusion surgery on CBF and WMD in animals with UCCAO. **A**, **Upper**: Representative images of CBF of mice from the sham, UCCAO, and reperfusion groups; **Lower**: Quantitative data of CBF ratio from different groups. **B**, **Upper:** Representative images of DTI from different grouped mice; **Lower**: FA values of DTI from the hypoperfusion and central CC regions. **C**, **Left**: Representative images of CC LFB staining in different groups; **Right**: The severity score of white matter lesion using LFB staining in 3 groups. **D**, **Left**: Representative images of TEM on the white matter from different mice; **Right**: The percentage of myelinated axons in different groups. **E**, **Upper**: Representation of immuno‐blots for MBP, SMI32, and OSP expressions in different groups. **Lower**: Bar graph of densitometric analysis. n=3–6. The *P* values were calculated using Student's *t* test and ANOVA. CBF indicates cerebral blood flow; CC, corpus callosum; DTI, diffusion tensor imaging; FA, fractional anisotropy; LFB, luxol fast blue; MBP, myelin basic protein; OSP, oligodendrocyte specific protein; SMI32, non phosphorylated neurofilament heavy chain; TEM, transmission electron microscope; UCCAO, unilateral carotid common artery occlusion; WMD, white matter damage; and WML, white matter lesion.

To investigate the changes in CBF in vivo, we used a Doppler flowmeter and laser speckle imaging techniques (Figure 4A). The baseline CBF ratios in the UCCAO group were significantly lower than those in the sham group. However, CBF in the hypoperfused regions was significantly upregulated after reperfusion (*P*<0.01).

To assess whether reperfusion reduces WMD in vivo, we performed rodent DTI. As shown in Figure 4B, UCCAO led to significant reductions in FA values in both the hypoperfusion and central corpus callosum regions. Conversely, reperfusion significantly improved the FA values in both the hypoperfused and central corpus callosum regions (*P*<0.01).

Oligodendrocytes, the sole myelinating cells of the central nervous system, are closely associated with WMD and CI.^21^ To assess myelin sheath damage, we performed corpus callosum luxol fast blue staining in mice. As shown in Figure 4C, UCCAO induced inflammation and nerve swelling, and reperfusion surgery markedly alleviated these injuries. The severity score of white matter lesions quantitatively confirmed that reperfusion reduced UCCAO‐induced upregulation of white matter lesions severity scores (*P*<0.05).

To directly observe the ultrastructural structure of the white matter in the different groups, corpus callosum specimens were harvested and observed using transmission electron microscope. As shown in Figure 4D, sham mice showed healthy and intact sheath plates and mitochondrial structures. However, the sheath plates were thickened, broken, and silked and disappeared after UCCAO induction. Moreover, mitochondria in oligodendrocytes were edematous in the UCCAO group. However, reperfusion significantly alleviated the UCCAO‐induced changes. Quantitatively, the UCCAO‐induced downregulation of myelinated axons was significantly alleviated by reperfusion (*P*<0.05). To further evaluate the changes in WMD at the protein level, we used western blotting to measure the MBP (myelin basic protein), SMI32 (nonphosphorylated neurofilament heavy chain), and OSP (oligodendrocyte‐specific protein) levels in the ipsilateral white matter tissues (Figure 4E). The protein levels of MBP, SMI32, and OSP were significantly upregulated in the reperfusion group compared with those in the UCCAO group (*P*<0.05).

### Reperfusion Operation Altered Ipsilateral Brain White Matter Transcriptional Profile

**Figure 5:**
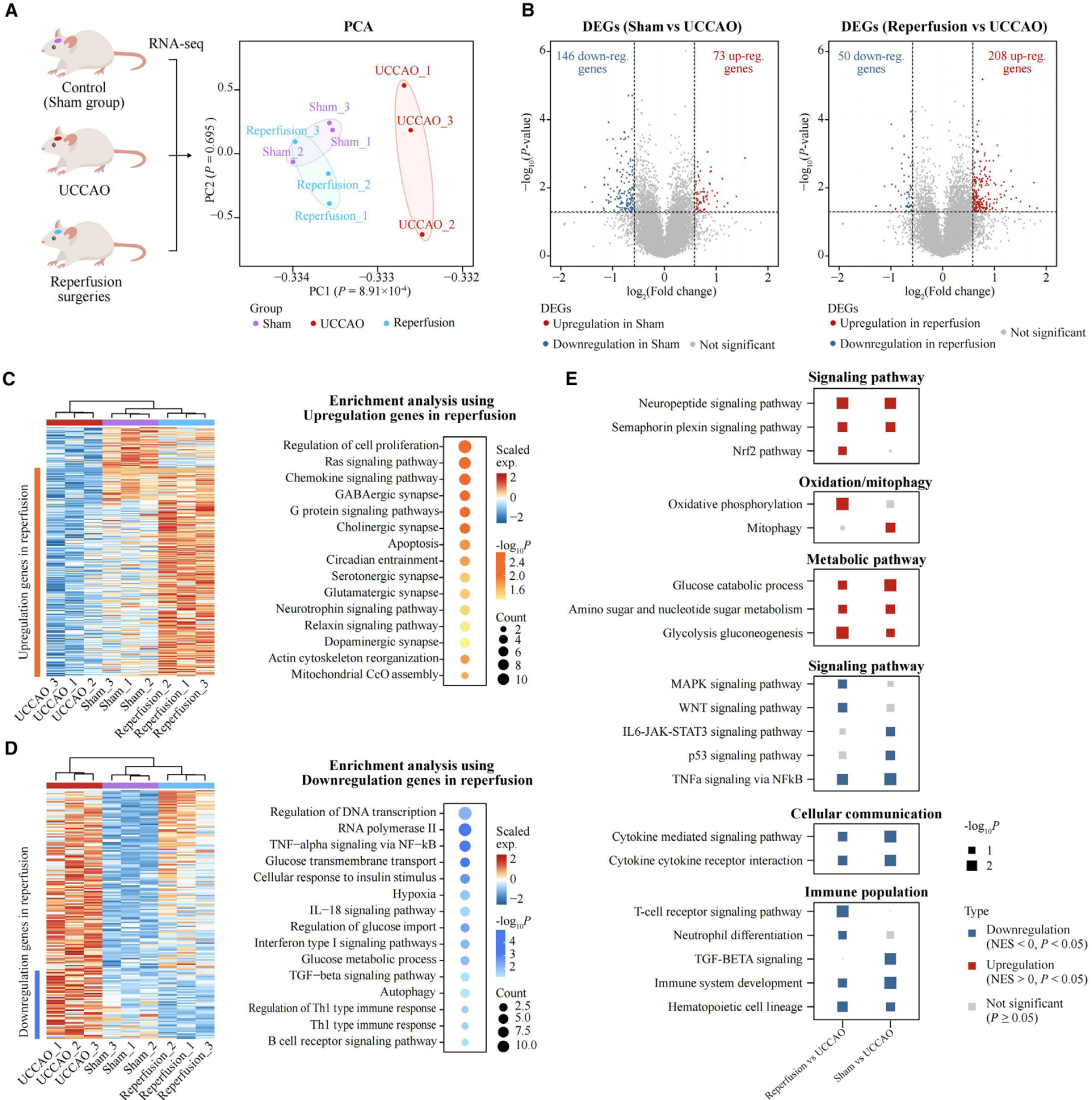
Transcriptome‐wide RNA‐sequencing assays to identify key pathways of reperfusion operation altered ipsilateral brain white matter transcriptional profile. The RNA‐seq was conducted at 7 d after reperfusion surgery. **A**, PCA plot shows the expression characteristics of 3 groups: sham, UCCAO, and reperfusion. n=3. The *P* values were calculated using ANOVA. **B**, Volcano plot illustrating DEGs between sham group and UCCAO group, as well as between the reperfusion group and the UCCAO group. Red dots represent upregulated genes, and blue dots represent downregulated genes. The *P* values were calculated using the R package limma. Two‐sided *P* values were calculated. **C**, Heatmap plot illustrating upregulated DEGs in both the sham and reperfusion groups. Gene ontology enrichment analysis highlights the KEGG pathways enriched using the upregulated genes between the reperfusion group and the UCCAO group. Pathways with *P* values <0.05 were illustrated. **D**, Heatmap plot illustrating down‐regulated DEGs in sham and reperfusion groups. Gene ontology enrichment analysis showing the KEGG pathways enriched using the downregulated genes between reperfusion group and UCCAO group. Pathways with *P* values <0.05 were illustrated. **E**, GSEA plot genes shows upregulated and downregulated pathways between sham group and UCCAO group, and reperfusion group and UCCAO group. Genes were ranked based on the fold change between the sham group and the UCCAO group, as well as the reperfusion group and the UCCAO group. The *P* value was calculated using GSEA. A 2‐sided *P* value was calculated. DEGs indicates differentially expressed genes; GSEA, gene set enrichment analysis; IL‐18, interleukin‐18; JAK/STAT, Janus kinase/signal transducers and activators of transcription; KEGG, Kyoto Encyclopedia of Genes and Genomes; MAPK, mitogen‐activated protein kinase; NF‐κB, nuclear factor‐kappa; NES, normalized enrichment score; PCA, principal component analysis; TGF‐β, transforming growth factor beta; TNF‐α, tumor necrosis factor alpha; and UCCAO, unilateral carotid common artery occlusion.

To explore the pivotal pathways following reperfusion, we performed RNA sequencing of tissue samples obtained from the ipsilateral brain white matter of the mouse model (Figure 5A). Principal component analysis revealed that, compared with the UCCAO group, the transcriptional profile of the reperfusion group closely resembled that of the control group (sham group, Figure 5A). Differential gene expression analysis was conducted separately between the sham and UCCAO groups and between the reperfusion and UCCAO groups using the R package limma (Figure 5B). Genes found to be significantly upregulated in the reperfusion group (log2 fold change >0.58 and *P*<0.05) were notably enriched in pathways associated with apoptosis, RAS signaling, and various cellular signaling pathways, including G protein‐coupled receptor and chemokine signaling pathways (Figure 5C). Conversely, genes that were significantly downregulated in the reperfusion group were associated with pathways related to immune cells and hypoxia (Figure 5D). We subsequently conducted gene set enrichment analysis to explore overall changes across the 3 groups. Notably, the NRF2 pathway was significantly upregulated in the reperfusion group, along with pathways associated with oxidative phosphorylation and mitophagy, compared with the UCCAO group. Moreover, metabolic‐related pathways, including glycolysis, gluconeogenesis, amino sugar and nucleotide sugar metabolism, and glucose catabolic processes, were upregulated in both the reperfusion and sham groups, indicating a potential restoration of brain function (Figure 5E). Regarding downregulated pathways, we observed a decrease in signaling associated with WNT and JAK–STAT (Janus kinase/signal transducers and activators of transcription), as well as p53 pathways and TNF‐α (tumor necrosis factor alpha) signaling via NF‐κB (nuclear factor‐kappa B), compared with the UCCAO group (Figure 5E). Additionally, pathways related to cellular communication and immune cells were downregulated, indicating potential vascular inhibition following reperfusion (Figure 5E).

### CEA Changed White Blood Cell Counts and Composition in Patients With CAS

**Figure 6:**
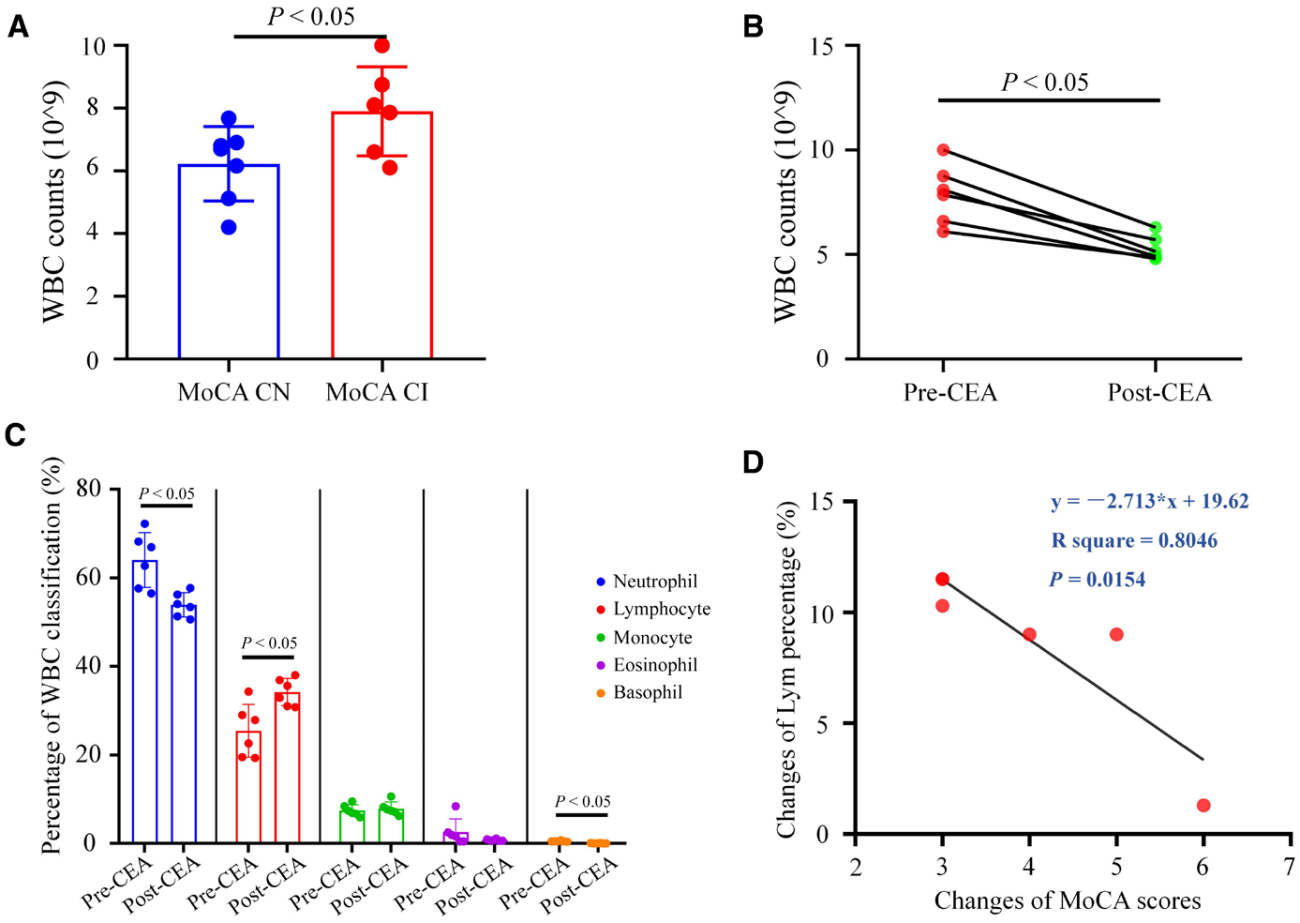
The effects of CEA on WBC in patients with CAS. **A**, WBC counts in patients with preoperative MoCA CN and CI with CAS. **B**, WBC counts in patients with preoperative MoCA CI with CAS before and 12 mo after CEA. **C**, The percentage of different WBC in patients with preoperative MoCA CI with CAS before and 12 mo after CEA. **D**, The correlation between the changes of MoCA scores and basophil percentage in patients with preoperative MoCA CI with CAS before and 12 mo after CEA. n=6–7. The *P* values were calculated using Student's *t* test and Pearson's correlation coefficients. CAS indicates carotid artery stenosis; CEA, carotid endarterectomy; CI, cognitive impairment; CN, cognitive normal; MoCA, Montreal Cognitive Assessment; and WBC, white blood cell.

Our transcriptional data indicate the potential role of cellular communication and immune cell populations in CAS. Because of ethical concerns and difficulty in obtaining brain specimens, we analyzed the peripheral white blood cell (WBC) count preoperatively and 1 year postoperatively. After 1 year, all WBC counts were decreased after CEA (*P*<0.01, Figure S3). Specifically, patients with MoCA CI exhibited significantly higher WBC counts than those in patients who were CN pre‐CEA (*P*<0.05, Figure 6A). However, no significant difference was found between patients with CI and CN using the MMSE pre‐CEA (*P*>0.05, Figure S4A). Also, no significance were found between pre‐CEA and post‐CEA in patients with CN rather than CI (Figure S4B,C). Remarkably, for patients with MoCA CI, CEA significantly downregulated WBC counts a year later (*P*<0.05, Figure 6B). In particular, CEA induced significant downregulation of the percentage of neutrophils and basophils and upregulation of the percentage of lymphocytes in patients with MoCA CI (*P*<0.05, Figure 6C). In addition, the improvement in MoCA scores was positively correlated with an elevated percentage of basophils within a year after CEA (*P*<0.05, Figure 6D). These findings suggest that WBC activation may contribute to cognitive progression in patients with CAS with MoCA‐related CI and that CEA surgery effectively reduced these blood inflammatory indices.

## DISCUSSION

Currently, CEA is well established as an effective method for treating CAS. However, the pathophysiological mechanisms underlying CAS and CEA are poorly understood.^22^ Several animal models of CCH have been established, from rodents to primates, which aid in the understanding of the phenotypic and pathophysiological mechanisms. Despite the abundance of available data, CCH lacks an operative definition and can be caused by various conditions, including CAS, chronic heart failure, hypotension, and atherosclerosis. Moreover, existing CCH animal models are mainly induced by the bilateral common carotid artery stenosis, bilateral common carotid artery occlusion, asymmetric common carotid artery surgery, and 3‐vessel occlusion.^23^ However, epidemiological data have shown that the incidence of unilateral CAS is much higher than bilateral CAS.^24^, ^25^ Thus, the existing CCH animal models cannot effectively replicate CAS. Our UCCAO model was established by ligating the unilateral common carotid artery, which was developed from the bilateral common carotid artery occlusion model by Yoshizaki et al.^26^ This model applies the characteristics of mouse posterior communicating artery dysplasia and causes CCH in the ipsilateral cerebral hemisphere by ligating 1 side of the common carotid artery. Therefore, we modified the model by ligating and loosening the common carotid artery with a fish thread. We found that this ligation model had a high survival rate (no mortality) and an obvious CBF decline, effectively mimicking unilateral CAS. Moreover, we performed a common carotid artery ligation loosening operation for the first time to simulate CEA in mice by restoring vessel stenosis. After ligation loosening, CBF in the poorly perfused regions was significantly improved, indicating that the model was successfully established.

The hybrid PET/MR system is a unique nuclear medicine technology for the simultaneous acquisition and analysis of both morphological and functional data. Given the lower soft tissue resolution of PET/CT, PET/MRI is considered a superior and more favorable imaging modality for brain diseases in clinical practice.^27^ Notably, we have reported that hybrid ^18^F‐fallypride PET/MRI is a promising technique for predicting dopamine agonists responses in patients with prolactinoma.^28^ Owing to its convenience and the stability of ^18^F‐FDG, it has traditionally been used as a PET tracer to study brain metabolism. Yoshida et al.^29^ used ^18^F‐FDG PET/CT to determine the effects of CEA on brain metabolism. However, this study was conducted using PET/CT, which cannot show detailed anatomical characteristics in both the hypoperfusion and whole‐brain subdivided regions when compared with PET/MRI. Moreover, it does not have the function of an MRI, which can integrate 3D ASL and DTI examinations. More recently, Cui et al.^30^ reported that simultaneous hybrid ^18^F‐FDG PET/MRI allows for the simultaneous analysis of brain hemodynamic and metabolic status in patients who underwent superficial temporal artery‐middle cerebral artery bypass surgery for symptomatic unilateral ischemic cerebrovascular disease. Herein, we employed a combination of ASL/DTI MRI and ^18^F‐FDG PET to evaluate CBF, metabolism, and white matter nerve fibers, which have proven to be essential and effective tools in CAS.

Severe CAS (≥75%) was closely associated with CI, even though these patients had no brain infarction on MRI. Thus, asymptomatic CAS is an independent risk factor of CI.^31^ Currently, patients with CAS who undergo CEA or carotid artery stenting are classified as the entire experimental group in most cognitive assessment studies. Regarding simple CEA treatment, there is significant inconsistency in the literature regarding the effects of CEA on cognitive function. Aceto et al.^32^ reported that up to 20% of the postoperative cognitive decline occurred in patients with CAS who underwent CEA. However, this meta‐analysis and Paraskevas^9^ commented that less than 6 months after CEA may produce erroneous and unreliable results in cognitive assessments. Our study indicated that patients with severe CAS showed a slight cognitive decline on day 4 post‐CEA, which was not significant. This phenomenon may be linked to intraoperative temporary hypoperfusion and postoperative hyperperfusion, which lead to hypoxic–ischemic injury of neurons following CEA in the short term.^33^, ^34^ At postoperative 6 months, both the MMSE and MoCA scores showed increasing trends, although with no significant differences. These results are consistent with those of another study in China.^34^ Our 3D ASL data revealed that CBF at 6 months following CEA markedly improved compared with that before CEA, indicating that this degree of quantitative CBF change was not sufficient to support qualitative changes in cognitive improvement. In line with Watanabe^35^ and Ghogawala,^36^ our study also indicated that patients had significantly improved cognitive function 12 months after CEA. However, this improvement was limited to patients with preoperative CI and the use of MoCA measurements. The reason long‐term cognitive improvement was seen only in MoCA but not in MMSE scores may be that MMSE is more suitable for mass screening, whereas MoCA is more suitable for identifying early CI, including mild CI and dementia. Compared with MoCA, MMSE is less sensitive with a narrow range of detection and may not detect mild cognitive improvement. These data suggest that CEA may lead to improved cognitive function in the long term, especially in patients with severe cognitive dysfunction before surgery.

Cognition is precisely titrated by multiple physiological mechanisms characterized by complex synergism, integration, and protective redundancy. Prior studies by Lattanzi et al.^37^ showed the existence of an interrelationship between hemodynamic and cognition, suggesting one potential mechanism of cognitive improvement after CEA may be attributed to the enhancement of cerebral vasomotor reactivity. Another possible reason for the improvement in cognitive function with CEA may be the postoperative elevation of ipsilateral CBF postoperatively.^36^ Our preclinical and clinical data suggested that CEA significantly improves CBF, which is associated with cognitive improvement. In humans, CBF is regulated by several reflexive responses, including cerebral metabolism.^38^ Interestingly, we found for the first time that CEA not only improved glucose metabolism in hypoperfusion areas but also in remote regions, including the ipsilateral prefrontal lateral, prefrontal medial, sensorimotor, anterior cingulate, parietal inferior, and temporal lateral regions. This phenomenon is due to CEA‐induced ICA revascularization, which improves all downstream intracranial branch vessels such as the anterior and middle cerebral arteries. In addition, CEA improved the contralateral prefrontal, medial prefrontal, and lateral prefrontal metabolism. This may be caused by postoperative CBF elevation on the affected side, resulting in a compensatory increase in contralateral blood flow.^30^ Our results indicate the necessity and validity of CEA.

Growing evidence suggests that cellular damage mechanisms in cognitive impairment after ischemic stroke are partly attributable to WMD, which could connect to ischemic cores in remote regions as a consequence of anterograde and retrograde Wallerian degeneration.^39^ Clinically, DTI is used to observe the microstructural integrity and structural connections of the matter to further assess the severity of WMD after stroke. In the present study, we found that CEA effectively attenuated WMD, leading to cognitive improvement. Oligodendrocytes contribute to the restoration of damaged myelinated axons, which is a key step for remyelination and functional recovery in WMD.^21^ Preclinical studies have suggested that revascularization may alleviate CAS‐induced oligodendrocyte number reduction and damage, thereby attenuating WMD and improving cognitive function.

Principal component analysis data revealed that the transcriptional profile of the reperfusion group closely resembled that of the sham group when compared with the UCCAO group, indicating that revascularization surgery effectively attenuated UCCAO‐induced WMD and even restored brain function close to normal. Specifically, the observed transcriptional changes in the mouse model suggest that revascularization‐induced neuroprotective effects may be mediated by the upregulation of Nrf2 signaling, downregulation of inflammation, and activation of metabolic pathways. Nrf2 plays a central neuroprotective role by regulating the cellular redox homeostasis and inflammation.^40^ Inflammasomes trigger and construct a platform for the downstream inflammatory cascade. Among various inflammasomes, the NLRP3 (NOD‐like receptor protein 3) inflammasome is the most extensively characterized. NF‐κB activation is particularly required for the NLRP3 inflammasome priming, and vice versa, NLRP3 inflammasome can induce the activation of NF‐κB. Moreover, our transcriptional data revealed that mitophagy was impaired following UCCAO induction. We previously indicated that Nrf2 could trigger and enhance mitophagy to inhibit the NLRP3 inflammasome and NF‐κB activation, thereby alleviating subsequent neuroinflammation. Recently, abnormal metabolites were shown to trigger excessive NLRP3 inflammasome activation and pathological inflammation.^41^ Nrf2 effectively protects neuronal cells from metabolic damage by driving metabolic reprogramming.^42^ In addition, CEA, which is used in combination with compounds that target the crosstalk between Nrf2 and inflammation, may be a prospective breakthrough in CAS treatment. Indeed, our previous studies also suggested that exogenous administration of Nrf2 activators and NLRP3 inflammasome inhibitors, such as ghrelin^43^ and cordycepin,^44^ could significantly attenuate brain injury.

A transcriptional survey also indicated that revascularization inhibited cellular communication and immune cell populations in UCCAO. Interestingly, a previous study revealed altered transcriptomes in the brain and peripheral blood monocyte‐derived macrophages and neutrophils, which are closely associated with brain injury in experimental strokes. More recently, elevated peripheral blood inflammatory indices have been associated with cognitive dysfunction.^45^ Herein, we revealed that WBC activation may contribute to cognitive progression in patients with CAS and MoCA‐related CI and that CEA surgery effectively reduced these blood inflammatory indices.

However, this study had some limitations that deserve attention. First, the number of patients with CAS included was limited, partly because of the high cost of PET/MR imaging. Second, mice employed herein were young, whereas CAS usually occurs in older people. In further study, animals with different ages and sexes should be applied to reduce the translational pitfalls. Third, only FA is employed as the primary DTI biomarker; future studies could extend to other DTI metrics to gain a deeper understanding of the changes in white matter structure before and after CEA. Moreover, our mechanistic study was based on a transcriptional survey. Further confirmatory experiments and in‐depth mechanistic investigations of CEA‐induced neuroprotection in severe unilateral CAS should be conducted to identify new therapeutic opportunities.

## CONCLUSIONS

Herein, we revealed that CEA could effectively ameliorate cognitive dysfunction in patients with severe unilateral CAS and in animals by improving cerebral CBF and metabolism and alleviating WMD. Further mechanistic studies suggested that this neuroprotective effect is mediated through the upregulation of antioxidant signaling and metabolic pathways, coupled with the inhibition of neuroinflammation, cellular communication, and immune cell population signaling via a transcriptional survey.

## Sources of Funding

This work was supported by the National Natural Science Foundation of China (82201616, 82101532, 82200153) and Interdisciplinary Program of Shanghai Jiao Tong University (YG2022QN008).

## Disclosures

None.

## Supporting information

Tables S1–S2
Figures S1–S4
