# Supplementary material for: Carotid Endarterectomy Ameliorates Cognitive Impairment in Clinical and Experimental Unilateral Carotid Artery Stenosis
Source: J Am Heart Assoc. 2025 Jan 16;14(2):e038388. doi: 10.1161/JAHA.124.038388 (PMC12054441; doi:10.1161/JAHA.124.038388)
Supplement: Supplementary file 1 — Tables S1–S2 Figures S1–S4 [file JAH3-14-e038388-s001.pdf]

Supplemental Material

Table S1. The detailed information of targeted antibodies.

Primary antibodies	catalog no.	dilution	Company
MBP	Ab40390	1 : 1,000	Abcam
SMI32	SMI-32P	1 : 1,000	BioLegend
OSP	Ab53041	1 : 1,000	Abcam
HRP Conjugated Goat anti-Rabbit IgG Goat Polyclonal Antibody	HA1001	1 : 5,000	Hua An
HRP Conjugated Goat anti-Mouse IgG Goat Polyclonal Antibody	HA1006	1 : 5,000	Hua An
β-actin	ab8227	1 : 5,000	Abcam

Table S2. The effects of CEA on cognitive function in patients with CAS at different time points.

Patients	MMSE				MoCA			
	Pre-CEA	Post-CEA 4d	Post-CEA 6m	Post-CEA 12m	Pre-CEA	Post-CEA 4d	Post-CEA 6m	Post-CEA 12m
1#	28	27	29	30	27	26	29	30
2#	18	20	26	28	20	19	25	24
3#	18	20	23	23	18	18	24	24
4#	22	23	28	28	22	22	26	27
5#	28	30	29	27	26	28	28	28
6#	30	30	30	30	28	27	28	30
7#	26	24	29	30	26	23	28	28
8#	30	30	30	30	30	29	30	30
9#	25	17	26	26	24	21	27	25
10#	29	27	30	30	27	28	29	27
11#	29	28	30	25	26	27	28	24
12#	26	25	27	28	24	24	27	29
13#	27	25	25	25	25	24	28	27

CEA: carotid endarterectomy; MMSE: Mini-Mental State Examination; MoCA: Montreal cognitive assessment.

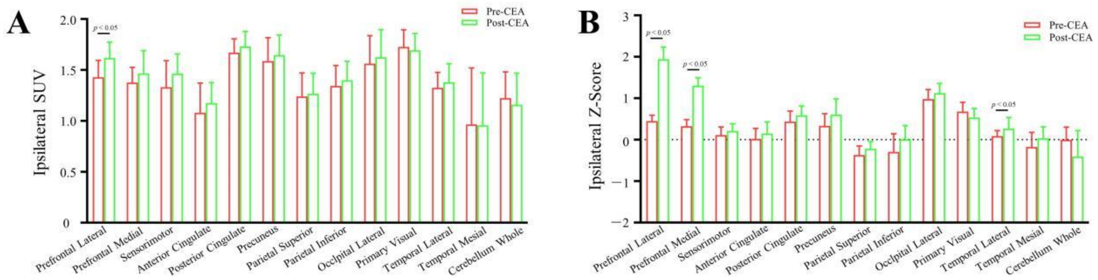

Figure S1. The effects of CEA on contralateral cerebral metabolism in patients with CAS. (**A**) SUVmean data before and after CEA in the whole contralateral brains. (**B**) Z-score data before and after CEA in the whole contralateral brains. n = 13. The *p*-values were calculated using Student's *t*-test. CEA: carotid endarterectomy; CAS: carotid artery stenosis; SUV: standardized uptake value.

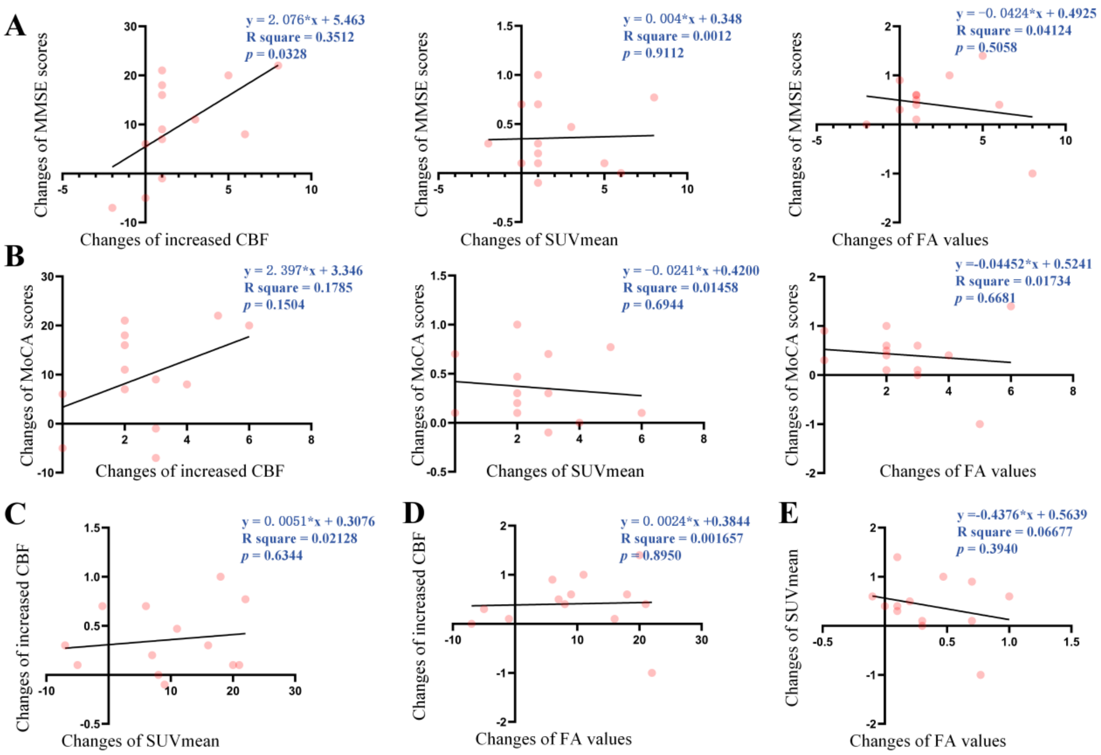

Figure S2. The correlations among the cognition, CBF, and cerebral metabolism pre- and post-CEA in patients with CAS. (**A**) The correlations between the CEA-induced changes of MMSE scores and CBF, SUVmean, and FA values. (**B**) The correlations between the CEA-induced changes of MoCA scores and CBF, SUVmean, and FA values. (**C**) The correlations between the CEA-induced changes of CBF and SUVmean. (**D**) The correlations between the CEA-induced changes of CBF and FA values. (**E**) The correlations between the CEA-induced changes of SUVmean and FA values. n = 13. The *p*-values were calculated using the Pearson's correlation coefficients. CEA: carotid endarterectomy; CAS: carotid artery stenosis; MMSE: Mini-Mental State Examination; MoCA: Montreal cognitive assessment; CBF: cerebral blood flow; SUV: standardized uptake value; FA: fractional anisotropy.

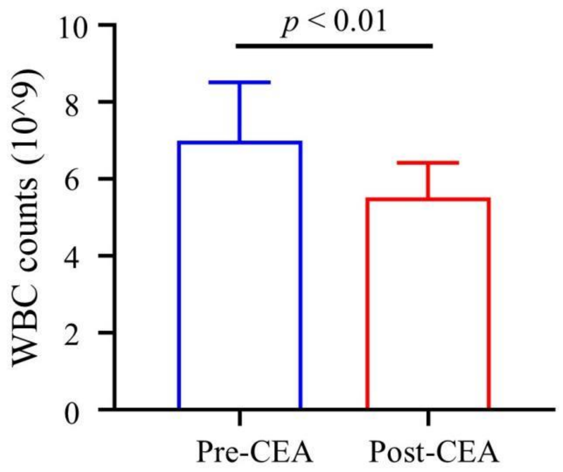

Figure S3. The effects of CEA on WBC in patients with CAS. WBC counts before and after CEA in patients with CAS. n = 13. The *p*-values were calculated using Student's *t*-test. CEA: carotid endarterectomy; CAS: carotid artery stenosis; WBC: withe blood cell.

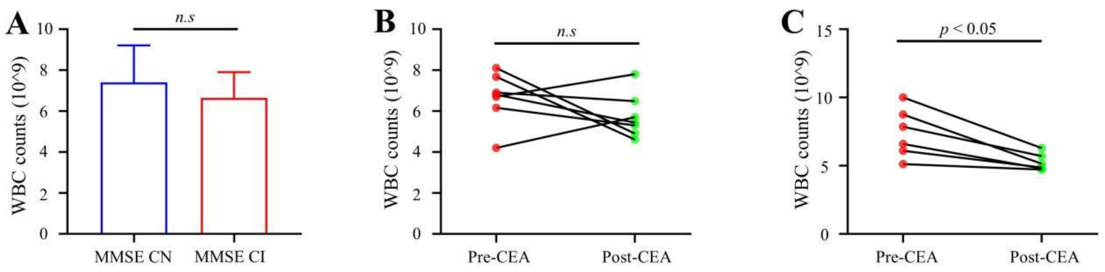

Figure S4. The effects of CEA on WBC in patients with CAS using MMSE scores. (**A**) WBC counts in preoperative MMSE CN and CI patients with CAS. (**B**) WBC counts in preoperative MMSE CN patients with CAS before and 12 m after CEA. (**C**) WBC counts in preoperative MMSE CI patients with CAS before and 12 m after CEA. n = 6 -7. The *p*-values were calculated using Student's *t*-test. CEA: carotid endarterectomy; CAS: carotid artery stenosis; MMSE: Mini-Mental State Examination; WBC: withe blood cell; n.s: no significance.
